# Retraction Note: Therapeutic effect of melatonin-loaded chitosan/lecithin nanoparticles on hyperglycemia and pancreatic beta cells regeneration in streptozotocin-induced diabetic rats

**DOI:** 10.1038/s41598-024-59109-0

**Published:** 2024-04-15

**Authors:** Habiba Alaa, Mariam Abdelaziz, Maryam Mustafa, Mustafa Mansour, Salma Magdy, Salma Mohsen, Yomna El-Karamany, Alyaa Farid

**Affiliations:** 1 Biotechnology/Biomolecular Chemistry Program, Faculty of Science, Cairo University, Giza, Egypt; 2 Immunology Division, Biotechnology Department, Faculty of Science, Cairo University, Giza, Egypt

Retraction of: *Scientific Reports* 10.1038/s41598-023-36929-0, published online 30 June 2023

The Editors have retracted this Article.

After publication of this Article, concerns with some of the data were brought to the attention of the Editors. Specifically:

- In Fig. 1G, several panels appear to show highly similar noise patterns, despite presenting results for different samples.
- In Fig. 7, there is an area of apparent overlap between panels N and Q. Panel R also appears to contain repetitive elements.
- In Fig. 8, there is an area of apparent overlap between panels H, J, and K. Panel B also appears to contain some repetitive elements.

The Authors were not able to provide raw data to alleviate these concerns. The Editors therefore no longer have confidence in the results and conclusions presented.

Alyaa Farid did not explicitly state whether or not they agree with this retraction. The Editors were unable to confirm contact details for Salma Magdy and Yomna El Karamany. The remaining authors did not respond to correspondence from the Editors about this retraction.

